# Oxylipin secretion by human CD3^+^ T lymphocytes *in vitro* is modified by the exogenous essential fatty acid ratio and life stage

**DOI:** 10.3389/fimmu.2023.1206733

**Published:** 2023-06-14

**Authors:** Johanna von Gerichten, Annette L. West, Nicola A. Irvine, Elizabeth A. Miles, Philip C. Calder, Karen A. Lillycrop, Graham C. Burdge, Barbara A. Fielding

**Affiliations:** ^1^ School of Chemistry and Chemical Engineering, Faculty of Engineering and Physical Sciences, University of Surrey, Guildford, Surrey, United Kingdom; ^2^ School of Human Development and Health, Faculty of Medicine, University of Southampton, Southampton, Hampshire, United Kingdom; ^3^ NIHR Southampton Biomedical Research Centre, University Hospital Southampton NHS Foundation Trust and University of Southampton, Southampton, Hampshire, United Kingdom; ^4^ Centre for Biological Sciences, Faculty of Natural and Environmental Sciences, University of Southampton, Southampton, Hampshire, United Kingdom; ^5^ Department of Nutritional Sciences, Faculty of Health and Medical Sciences, University of Surrey, Guildford, Surrey, United Kingdom

**Keywords:** immunosenescence, oxylipin, eicosanoid, lipid metabolism, T lymphocytes

## Abstract

Immune function changes across the life stages; for example, senior adults exhibit a tendency towards a weaker cell-mediated immune response and a stronger inflammatory response than younger adults. This might be partly mediated by changes in oxylipin synthesis across the life course. Oxylipins are oxidation products of polyunsaturated fatty acids (PUFAs) that modulate immune function and inflammation. A number of PUFAs are precursors to oxylipins, including the essential fatty acids (EFAs) linoleic acid (LA) and α-linolenic acid (ALA). LA and ALA are also substrates for synthesis of longer chain PUFAs. Studies with stable isotopes have shown that the relative amounts of LA and ALA can influence their partitioning by T lymphocytes between conversion to longer chain PUFAs and to oxylipins. It is not known whether the relative availability of EFA substrates influences the overall pattern of oxylipin secretion by human T cells or if this changes across the life stages. To address this, the oxylipin profile was determined in supernatants from resting and mitogen activated human CD3^+^ T cell cultures incubated in medium containing an EFA ratio of either 5:1 or 8:1 (LA : ALA). Furthermore, oxylipin profiles in supernatants of T cells from three life stages, namely fetal (derived from umbilical cord blood), adults and seniors, treated with the 5:1 EFA ratio were determined. The extracellular oxylipin profiles were affected more by the EFA ratio than mitogen stimulation such that n-3 PUFA-derived oxylipin concentrations were higher with the 5:1 EFA ratio than the 8:1 ratio, possibly due to PUFA precursor competition for lipoxygenases. 47 oxylipin species were measured in all cell culture supernatants. Extracellular oxylipin concentrations were generally higher for fetal T cells than for T cells from adult and senior donors, although the composition of oxylipins was similar across the life stages. The contribution of oxylipins towards an immunological phenotype might be due to the capacity of T cells to synthesize oxylipins rather than the nature of the oxylipins produced.

## Introduction

Progression through the life course is accompanied by changes in the immune system from prenatal immune tolerance to immune competence in adulthood, and immunosenescence and inflammaging in later years; the latter are characterized by increased susceptibility to cancer and autoimmune diseases, and reduced ability to respond to pathogens and vaccines (1, 2). Immunosenescence involves modifications in the numbers and activity of various T cell populations and is accompanied by chronic low-grade inflammation (3, 4). T cell activation, differentiation, and migration can be influenced by the polyunsaturated fatty acid (PUFA) composition of the cell membrane (5, 6). For example, PUFAs have structural roles in the cell membrane and can be used as substrates for further synthesis of immunoactive lipids (e.g. eicosanoids and other oxylipins). We have shown previously that the fatty acid composition of human T lymphocytes changes across the life course (7). Changes to the PUFA content of T lymphocytes may contribute to differences in production of lipid mediators, such as oxylipins, across life stages.

Oxylipins are oxidation products of PUFAs released from membrane phospholipids by phospholipase A2 (PLA2)), some of which are lipid mediators with immunomodulatory actions (e.g. prostaglandins, leukotrienes and lipoxins) (8). The essential fatty acids (EFAs), linoleic acid (LA; 18:2n-6) and α-linolenic acid (ALA; 18:3n-3), have been shown to have beneficial effects on age-related metabolic conditions (9–14), possibly through the actions of their respective 18 carbon atom oxylipin products, namely hydroxyoctadecaenoic acids (HODEs) and hydroxyoctatrienoic acids (HOTrEs), respectively.

We have shown in T lymphocytes from healthy young adults that 9- and 13-HODE are synthesized preferentially from newly assimilated LA, and that 9- and 13-HOTrE are synthesized from newly assimilated ALA (15). This suggests that a rapid uptake of exogenous EFAs by activated cells changes the balance of intracellular substrates so that pre-existing cellular pools are outcompeted by newly assimilated fatty acids. The synthesis of these oxylipins was influenced in a reciprocal manner by the relative amounts of LA and ALA in the culture medium (the EFA ratio). Furthermore, we have shown that the fatty acid composition and ALA metabolism in human T lymphocytes differs between life stages (7) potentially contributing to the difference in immune function over the life course. Despite these recent observations, the pathways and patterns of oxylipin synthesis and how the PUFA composition of the cell membrane influence the oxylipin profile produced by cells and ultimately immune function (16) across the life course have not been studied in humans yet. Oxylipins are formed *via* oxidation of PUFAs by lipoxygenases, cytochrome P450s, cyclooxygenases or autoxidation (17). 9- and 13-HODE are lipoxygenase metabolites of LA that have anti-proliferative characteristics in general, and more specifically are agonists for the peroxisome proliferator-activated receptor (PPAR)-γ that has been shown to play a role in macrophage development and endothelial dysfunction (18–21). ALA-derived oxylipins are less well characterized than their n-6-derived counterparts and are generally viewed as less-inflammatory (22). HOTrEs, which are synthesized from ALA by lipoxygenase activity, have been reported to suppress M1-like macrophage development (23) as well as being PPAR agonists (24). Arachidonic acid (AA; 20:4n-6), eicosapentaenoic acid (EPA; 20:5n-3) and docosahexaenoic acid (DHA; 22:6n-3) (25) are precursors of a variety of oxylipins such as prostaglandins, leukotrienes and lipoxins, and E- and D-series resolvins (16, 22). Feeding young rats a high AA diet for 13 weeks increased the amounts of AA-derived prostaglandins in plasma while feeding EPA and DHA increased hydroxyeicosapentaenoic acid (HEPE) and hydroxydocosahexaenoic acid (HDHA) levels (26). Similar results where changing supply of a PUFA only resulted in oxylipins produced directly from that PUFA substrate were demonstrated in a model of primary human brain endothelial cells treated with either AA, EPA or DHA (27).

Although EFAs, besides being substrates for oxylipin synthesis, are substrates for longer chain PUFA synthesis, the effect of EFAs on EPA- and DHA-derived oxylipin synthesis has to the best of our knowledge not been studied. Additionally, EFAs and other PUFAs compete as substrates for the same oxidative enzymes leaving the question of substrate specificity to be determined. There are only few studies on oxylipin patterns in mouse serum and blood indicating distinct substrate preferences and substrate competitions after EFA and PUFA specific diets (28, 29). Consequently, there is evidence that EFA and PUFA availability can influence the synthesis of immunomodulatory lipid products such as oxylipins and, therefore, might influence immune function; however, the mechanisms behind those processes in humans are still unclear.

Because the oxidation of 20 and 22 carbon PUFAs and 18 carbon EFAs to their respective oxylipins is catalyzed by the same cytochromes and lipoxygenases, we hypothesized that modifying the EFA content of the culture medium can alter the pattern of oxylipins secreted by T lymphocytes irrespective of the PUFA precursor. Furthermore, we hypothesized that PUFA synthesis might be altered during different life stages and that the oxylipin pattern would reflect these differences. To test the influence of EFA content, CD3^+^ T cells from peripheral blood of healthy adults were incubated for 48 h in the presence or absence of concanavalin A (Con. A) in medium containing LA and ALA in two different EFA ratios (15). Furthermore, to investigate whether life stage modified the pattern of oxylipins, fetal T cells from umbilical cord blood and T cells from peripheral blood of healthy adults or healthy seniors were incubated with a fixed EFA ratio. Culture supernatants were collected and the oxylipin composition determined by LC-MS/MS.

## Methods

### Ethics statement, participants, and collection of blood samples

The study was reviewed and approved by the East of England - Cambridge Central Research Ethics Committee (approval number 19/EE/0096) and all participants gave written informed consent. Sample collection and T cell isolation and culture are described in detail elsewhere (15).

### Isolation and culture of CD3^+^ T cells from whole blood

Mononuclear cells were isolated from blood of 10 healthy adult female participants (aged 18-30 years), to compare two different EFA ratios, and from blood of 5 healthy senior adults (3 female, 2 male; aged 68-74 years), to compare the effect of life stage. CD3^+^ T cells were isolated from PBMCs by negative selection as described elsewhere (15). Isolated cells were washed with 10 ml of RPMI1640 (Sigma-Aldrich cat no R0883) containing autologous pooled heat-inactivated serum (10%, Sigma-Aldrich, H3667) and collected by centrifugation at 300 × g for 10 min at room temperature. Cryopreservation of cells was carried out as described elsewhere (15).

Due to the SARS-CoV-2 pandemic recruiting of adults and sample collection was stopped and alternative suitable cells were sourced commercially. Cryopreservation of cells was carried out as described elsewhere (30, 31). T cell culture was carried out essentially as described elsewhere (32) with cells being cultured in RPMI1640 medium containing 10% (v/v) heat-inactivated serum, penicillin (100 units/ml) and streptomycin (100ug/ml) under 5% CO_2_, and activated with Concanavalin A (Con.A; 10 μg/mL; Sigma-Aldrich) or cultured without mitogen stimulation for 48 h. T cells were collected from the culture dish by scraping and centrifuged at 300 x g. The medium was discarded and the cells were then washed once withice cold PBS (1.5 ml). The cells were centrifuged again, the PBS discarded and then the cell pellets were resuspended in 0.8ml of 0.9% (w/v) NaCl. To model different dietary EFA intakes, the fatty acid composition of the culture medium (including fatty acids derived from the serum supplement) was adjusted by addition of ALA and LA (both Sigma-Aldrich) to give an LA to ALA ratio (EFA ratio) of either 5:1 or 8:1 for 48h, which is representative of LA : ALA ratios in some European diets (33). LA concentration was maintained at 185 µM (mainly present in the added serum) and ALA concentration was altered according to the desired ratio. Supernatants were collected, snap-frozen in liquid nitrogen and stored at −80°C.

### Commercially acquired T lymphocytes and PBMCs

Cryopreserved CD3^+^ T cells were purchased (Catalog number 70024.1) from Stemcell Technologies UK Ltd (Cambridge, UK); these T cells were collected from 8 anonymous adult donors (aged 22-29 years; 3 females, 5 males) and 3 senior adults (aged 52-58 years; 1 female, 2 male) whose characteristics met the inclusion and exclusion criteria for the study and were used to compare different life stages. Umbilical cord blood mononuclear cells (n=8) were purchased from Stemcell Technologies (Catalog number 70007.1) and used to prepare fetal CD3^+^ T lymphocytes.

### Analysis of oxylipins in T cell supernatants by liquid chromatography-tandem mass spectrometry

The frozen supernatant from resting or mitogen-activated T cells was thawed and free oxylipins were isolated by solid phase extraction (SPE) (34) as described elsewhere (15). Briefly, the internal standards 17(*S*)-hydroxydocosa-4,7,10,13,15,19-hexaenoic-21,21,22,22,22-d_5_-acid ([d_5_]17-HDHA; 20 ng), 9α,11α,15S-trihydroxy-prost-13E-en-1-oic-17,17,18,18,19,19,20,20,20-d_9_-acid ([d_9_]PGF1α; 20 ng) and (±)14,15-dihydroxy-5Z,8Z,11Z-eicosatrienoic-16,16,17,17,18,18,19,19,20,20,20-d_11_-acid ([d11]14,15-DiHETrE; 20 ng) were added to the defrosted supernatants and protein precipitated with methanol. Samples were acidified with 10 μL 1M HCl and oxylipins purified using Oasis HLB (Waters) SPE cartridges (34), stored in 100 µL methanol/water 70:30 (v/v) at -20°C and analysed by liquid chromatography-tandem mass spectrometry (LC-MS/MS) within 48 h. Oxylipins were detected by multiple reaction monitoring (MRM) using an Acquity I-class and Xevo TQS UPLC-MS/MS system (Waters). Negative ESI parameters were: 2.4 kV capillary voltage, 40 V cone voltage, 600°C desolvation temperature, 1000 L/h desolvation flow, 150 L/h cone flow and 7 bar nebuliser pressure. MRM transitions are shown in **Supplementary Table 1**. The chromatography method is described elsewhere (15). Briefly, lipids were separated using a C18 column (Waters) with a C18 pre-column (Waters) at 40°C and a flow rate of 0.3 mL/min. The mobile phases for the linear gradient were A 80:20 (v/v) water/acetonitrile and B 75:25 (v/v) acetonitrile/methanol, both containing 0.02% (v/v) formic acid. The limit of detection (LOD), SPE recovery and inter-assay coefficient of variation are reported in **Supplementary Table 1**. Data were processed using MassHunter 4.0 (Waters).

### Analysis of fatty acids in T cells and culture medium by GC-MS

Total fatty acid composition was measured in T cells and medium by standard laboratory techniques as detailed previously (32). The fatty acid composition of the medium was verified for each experiment.

### Data analysis and statistics

Nomenclature of detected oxylipins is according to Lipid Maps Structure Database, https://www.lipidmaps.org/data/classification/LM_classification_exp.php) (35). Oxylipin concentrations were calculated relative to the internal standards [d5]17-HDHA for monohydroxy compounds, [d11]14,15-DiHETrE for dihydroxy compounds and [d9]PGF1α for trihydroxy compounds. Values were then corrected for supernatant volume, normalized to the number of T cells in the cell culture, background corrected for cell media and reported as pmol [alternatively mol x 10-12] oxylipin/106 T cells. Oxylipins with more than 50% signals above LOD in the total amount of samples detected were defined as valid and used for multivariate analysis with MetaboAnalyst 5.0 as ratios (fold change) of increased ALA levels (5:1 EFA ratio) to control western diet levels (8:1 EFA ratio) and fetal or senior life stage to adult life stage (https://www.metaboanalyst.ca/MetaboAnalyst/ModuleView.xhtml) (36). Data were log transformed and auto-scaled (mean-centered and divided by the standard deviation of each variable) before partial least squares – discriminant analysis (PLS-DA). Variable Importance in Projection (VIP) was calculated for each oxylipin. Oxylipins with VIP values>1.1 were selected for further statistical analysis. SPSS version 27 (IBM SPSS Statistics for Windows, Armonk, NY: IBM Corp) was used for linear mixed model analysis with mitogen activation and EFA ratio as repeated measure (unstructured covariance) and multiple comparison was corrected with the Sidak method. GraphPad Prism version 8.4.3 for Windows (GraphPad Software, San Diego, California USA) was used for one-way ANOVA and t-test.

## Results

**Figure 1:**
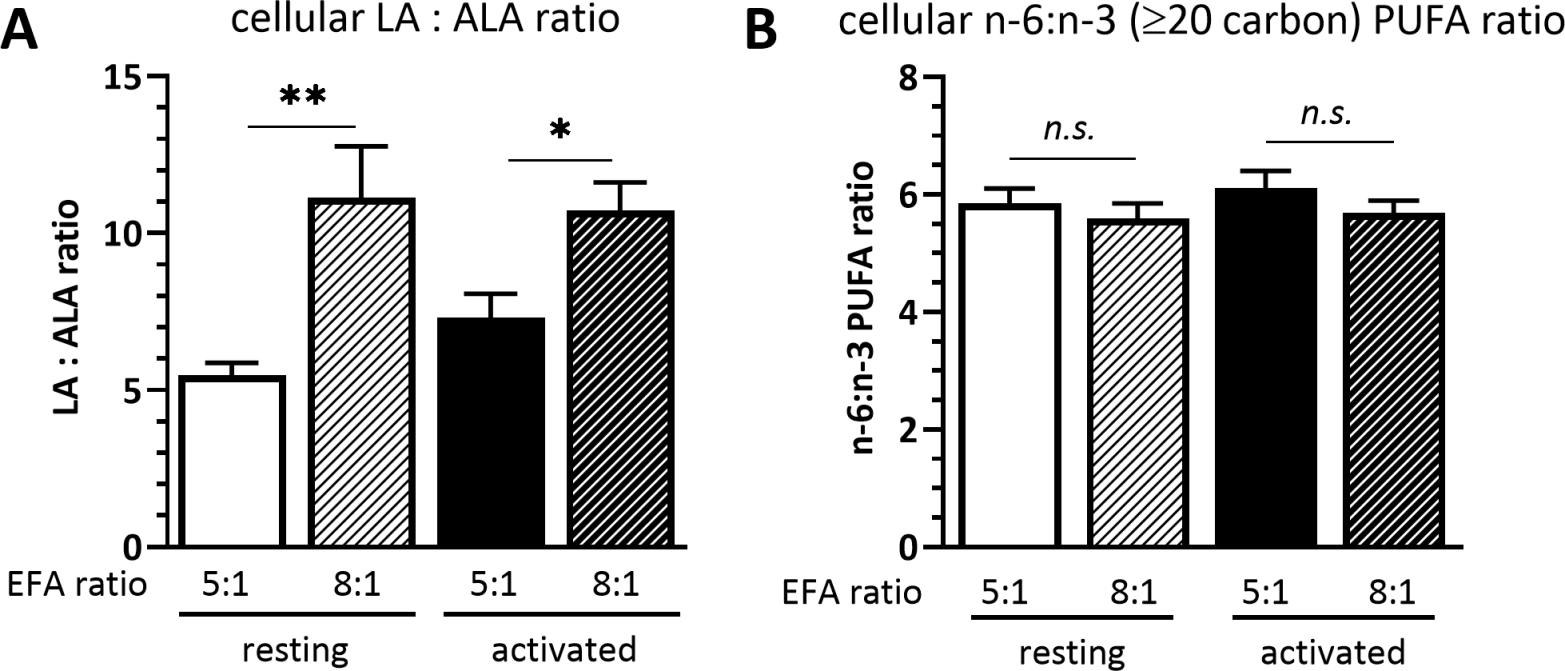
**(A)** Cellular LA : ALA ratio and **(B)** ≥C20 n-6:n-3 PUFA ratio of T cells after 48 h culture in medium containing a 5:1 or 8:1 EFA ratio (determined by GC-FID). Values are mean ± SEM, n=10 subjects (fresh blood donations). Paired Sidak multiple comparison (α=0.05), *<0.05, **<0.01, n.s, not significant.

The cellular LA to ALA ratio was significantly lower in cells cultured in medium with a 5:1 EFA ratio compared to an 8:1 EFA ratio, both in resting (5.5 ± 0.4 5:1 versus 11.1 ± 1.6 8:1 EFA ratio; P=0.004) and activated T cells (7.3 ± 0.7 5:1 versus 10.7 ± 0.9 8:1 EFA ratio; P = 0.038). The cellular n-6 to n-3 ratio calculated with the LA and ALA fatty acid metabolites eicosadienoic acid (20:2n-6), dihomo-γ-linolenic acid (20:3n-6), AA (20:4n-6) and docosatetraenoic acid (22:4n-6), as well as eicosatrienoic acid (20:3n-3), EPA (20:5n-3), docosapentaenoic acid (22:5n-3) and DHA (22:6n-3), respectively, but without including LA and ALA, was not changed according to the EFA ratio in the culture medium (**Figure 1**).

### Effect of mitogen activation and culture medium EFA ratio on the oxylipin profile of supernatants from T lymphocyte cultures (adult donors)

**Figure 2:**
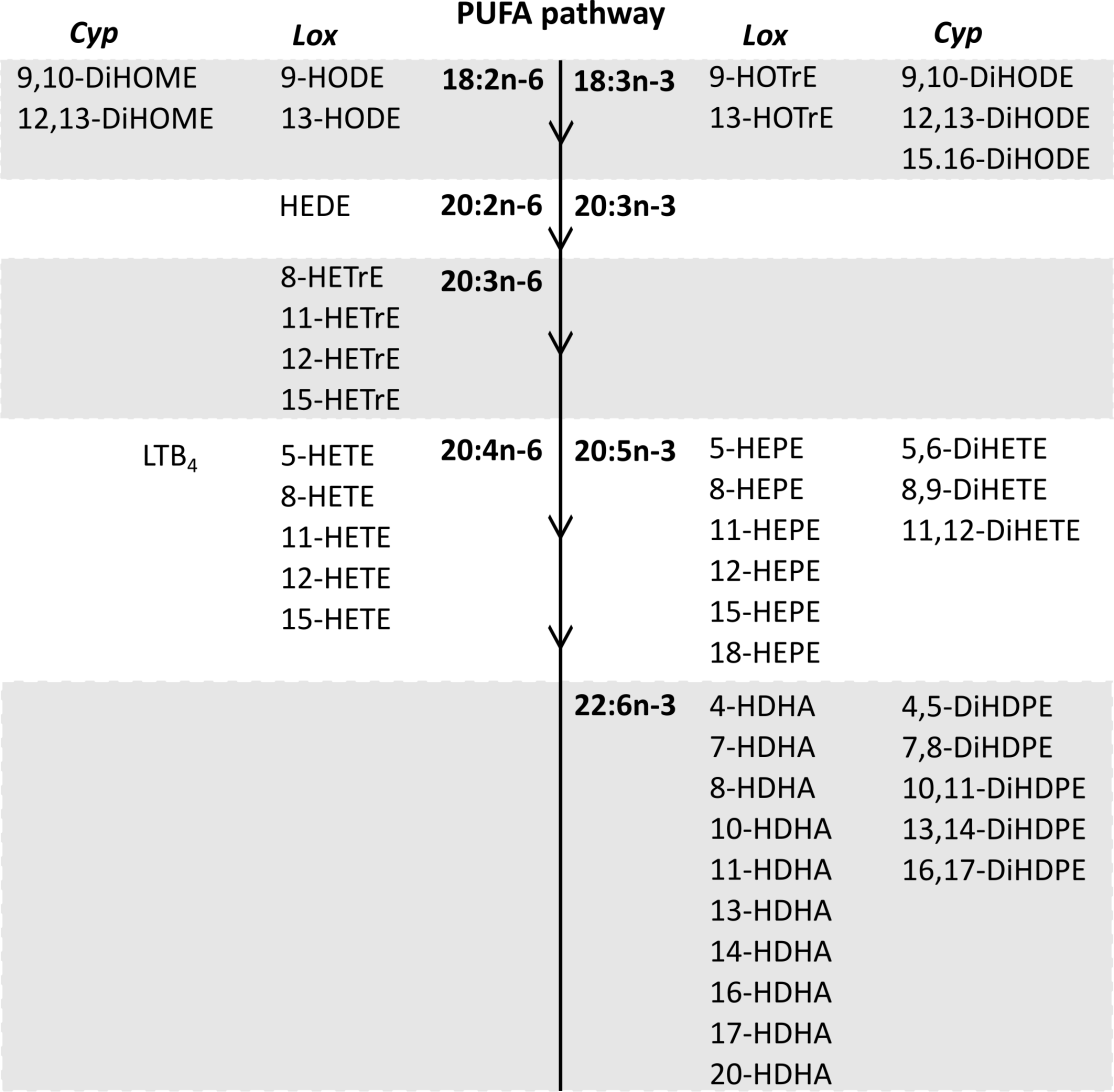
The n-6 and n-3 PUFA synthesis pathway in human T lymphocytes (32) and corresponding oxylipins from cytochrome P450 and lipoxygenase catalysed synthesis detected in CD3+ T cell supernatants after 48 h cell culture (PGE2 and PGD2 synthesis by the cyclooxygenase pathway are not shown). Lox, Lipoxygenase; Cyp, Cytochrome P450.

**Figure 3:**
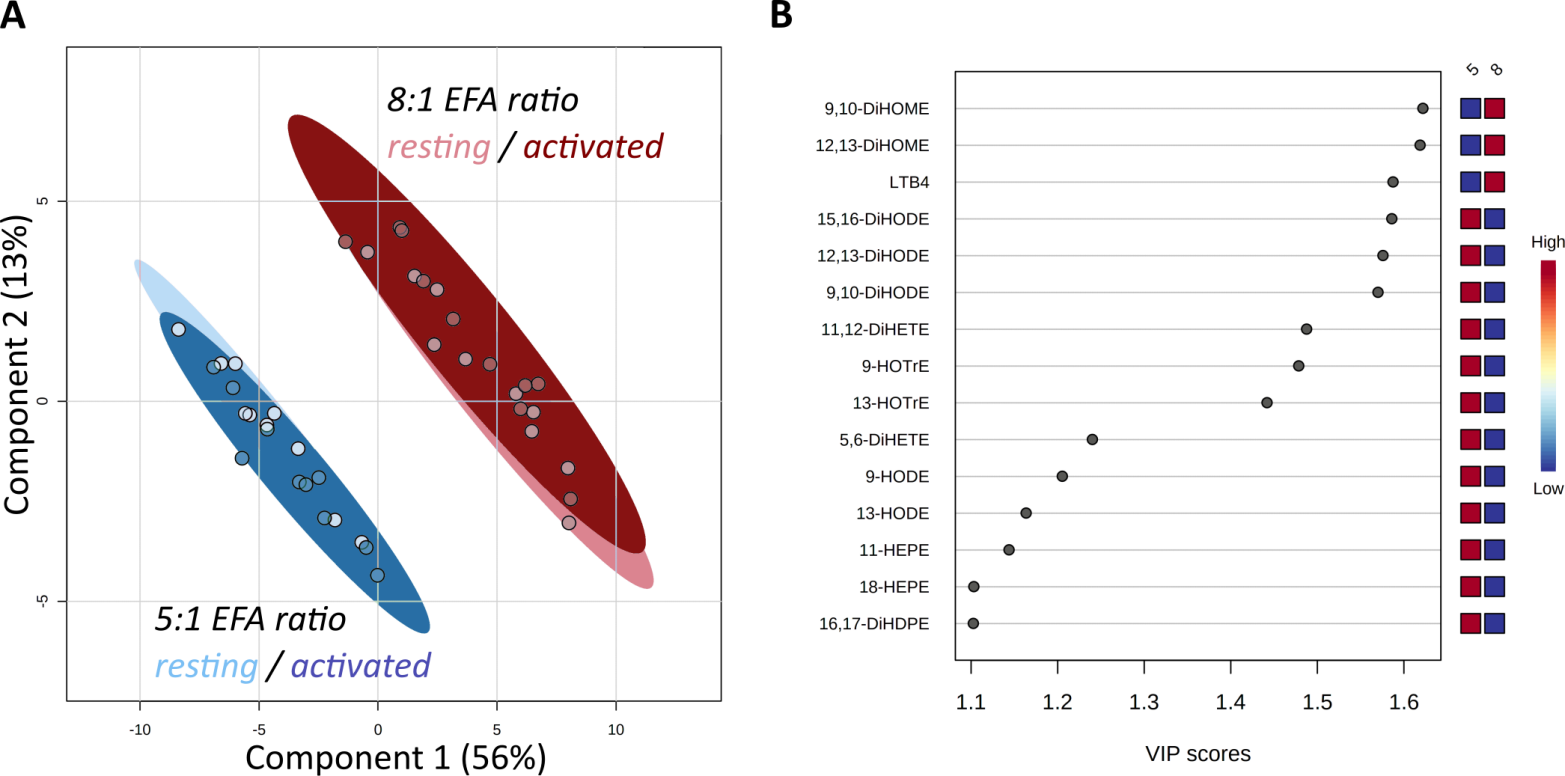
Score Plot from Partial Least Square-Discriminant Analysis (PLS-DA) of oxylipins in supernatants from CD3+ T-cells **(A)**. Main effect is shown by the separation based on the difference in EFA ratio with 5:1 (blue) versus 8:1 (red) for both resting (light) and activated (dark) T-cells. **(B)** Top 15 VIP compounds identified by PLS-DA. n=10 subjects (fresh blood donations).

Forty-seven oxylipins were detected consistently. These have been shown to be derived from LA, ALA, 20:2n-6, 20:3n-6 or 20:3n-3, AA, EPA, and DHA (16) (**Figure 2**). The concentration of one oxylipin (15-HETrE p=0.027, **Supplementary Table 2**) differed significantly only between culture supernatants from resting and activated T cell cultures, but the concentrations of nine oxylipins were affected by either activation and interaction between activation x EFA ratio or interaction between activation x EFA ratio only (**Supplementary Table 2**). The concentrations of thirty-one oxylipins were significantly different according to the EFA ratio, whilst the concentrations of six oxylipins did not differ significantly between culture conditions (**Table 1**, **Supplementary Table 2**). Mitogen stimulation did not significantly alter the oxylipin profile in supernatants of T cells maintained in medium containing either the 5:1 or 8:1 EFA ratio: the partial least square discriminant analysis (PLS-DA) score plot shows no difference for oxylipin profiles of resting and activated T cells (accuracy=0.75, R^2 ^= 0.44 and Q^2 ^= 0.15 5:1 EFA ratio, and accuracy=0.45, R^2 ^= 0.21 and Q^2^=-0.22 8:1 EFA ratio) (**Figure 3A**). The oxylipin profile in the supernatants of cells maintained in medium with a 5:1 EFA ratio differed compared to that from cultures with the 8:1 EFA ratio for both resting and activated T cells (accuracy=0.9, R^2 ^= 0.77 and Q^2 ^= 0.71 for resting, and accuracy=0.9, R^2 ^= 0.79 and Q^2 ^= 0.71 for activated; **Figure 3A**). Of the 47 oxylipins detected, 15 were identified as important features (PLS-DA) with a VIP score of more than 1.1 comparing 5:1 and 8:1 EFA ratios for both resting and activated T cells (**Figure 3B**, **Table 1**).

**Table 1:** Fold change as log pmol oxylipin per 10^6^ T cells (5:1/8:1 EFA ratio) of the top 15 VIP oxylipins in supernatants from adult donor T cell cultures.

pmol oxylipin / 10^6^ T cells	log (5:1/8:1) EFA ratio		P value				
	Resting	Activated	Activation	EFA ratio	Activation x EFA ratio	Resting 5:1 vs 8:1	Activated 5:1 vs 8:1
9-HODE	0.196 ± 0.012	0.214 ± 0.011	0.312	0.001	0.491	0.001	0.001
13-HODE	0.159 ± 0.010	0.204 ± 0.009	0.269	0.001	0.266	0.002	0.004
9,10-DiHOME	-1.505 ± 0.105	-1.484 ± 0.077	0.715	<0.001	0.778	<0.001	<0.001
12,13-DiHOME	-1.496 ± 0.107	-1.497 ± 0.074	0.838	<0.001	0.854	<0.001	<0.001
LTB_4_	-1.247 ± 0.080	-1.580 ± 0.323	0.058	<0.001	0.053	<0.001	0.001
9-HOTrE	0.529 ± 0.074	0.685 ± 0.073	0.237	<0.001	0.109	<0.001	<0.001
13-HOTrE	0.422 ± 0.054	0.564 ± 0.049	0.197	<0.001	0.119	<0.001	<0.001
9,10-DiHODE	0.698 ± 0.058	0.732 ± 0.032	0.167	<0.001	0.265	<0.001	<0.001
12,13-DiHODE	0.806 ± 0.062	0.881 ± 0.058	0.130	<0.001	0.124	<0.001	<0.001
15,16-DiHODE	0.785 ± 0.057	0.781 ± 0.039	0.278	<0.001	0.472	<0.001	<0.001
11-HEPE	0.198 ± 0.031	0.326 ± 0.036	0.779	<0.001	0.140	0.001	0.001
18-HEPE	0.292 ± 0.033	0.335 ± 0.032	0.399	<0.001	0.558	0.005	0.002
5,6-DiHETE	0.281 ± 0.027	0.229 ± 0.021	0.327	<0.001	0.518	<0.001	0.001
11,12-DiHETE	0.714 ± 0.085	0.654 ± 0.049	0.674	<0.001	0.717	<0.001	<0.001
17-HDHA	0.226 ± 0.018	0.147 ± 0.012	0.298	0.001	0.194	0.002	0.012

Values are mean ± SEM (n = 10 paired). Comparisons were done using a linear mixed model, and statistical significance was assumed at p < 0.05. Adjustment for multiple t-tests: Sidak method. List of oxylipins is continued in **Supplementary Table 2**.

### Effect of EFA ratio in the medium on individual (VIP) oxylipins in supernatants from activated cultures of T lymphocytes from adult donors

**Figure 4:**
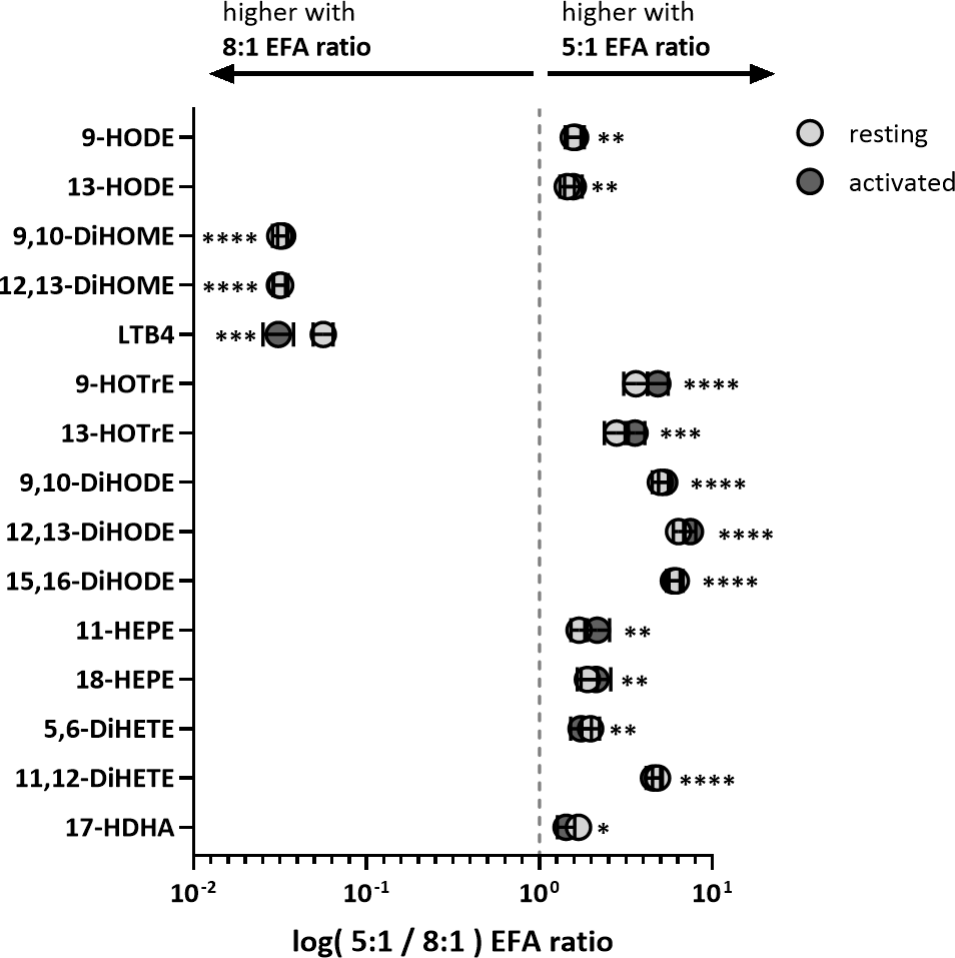
Fold change of VIP oxylipins in adult donor T cell supernatants identified by PLS-DA. Values are shown as mean log of pmol oxylipin per 10^6^ T cells from cultures treated with 5:1 versus 8:1 EFA ratio for both resting (light grey) and activated (dark grey) CD3+ T cells. Significances (SEM; black line) are shown for activated T cells (values for resting T cells are similar and shown in **Table 1**) and were adjusted using paired Sidak multiple comparison for EFA ratio in supernatant from either resting or activated T cells (n=10 subjects (fresh blood donations), α=0.05), *<0.05, **<0.01, ***<0.001 and ****<0.0001.

**Figure 5:**
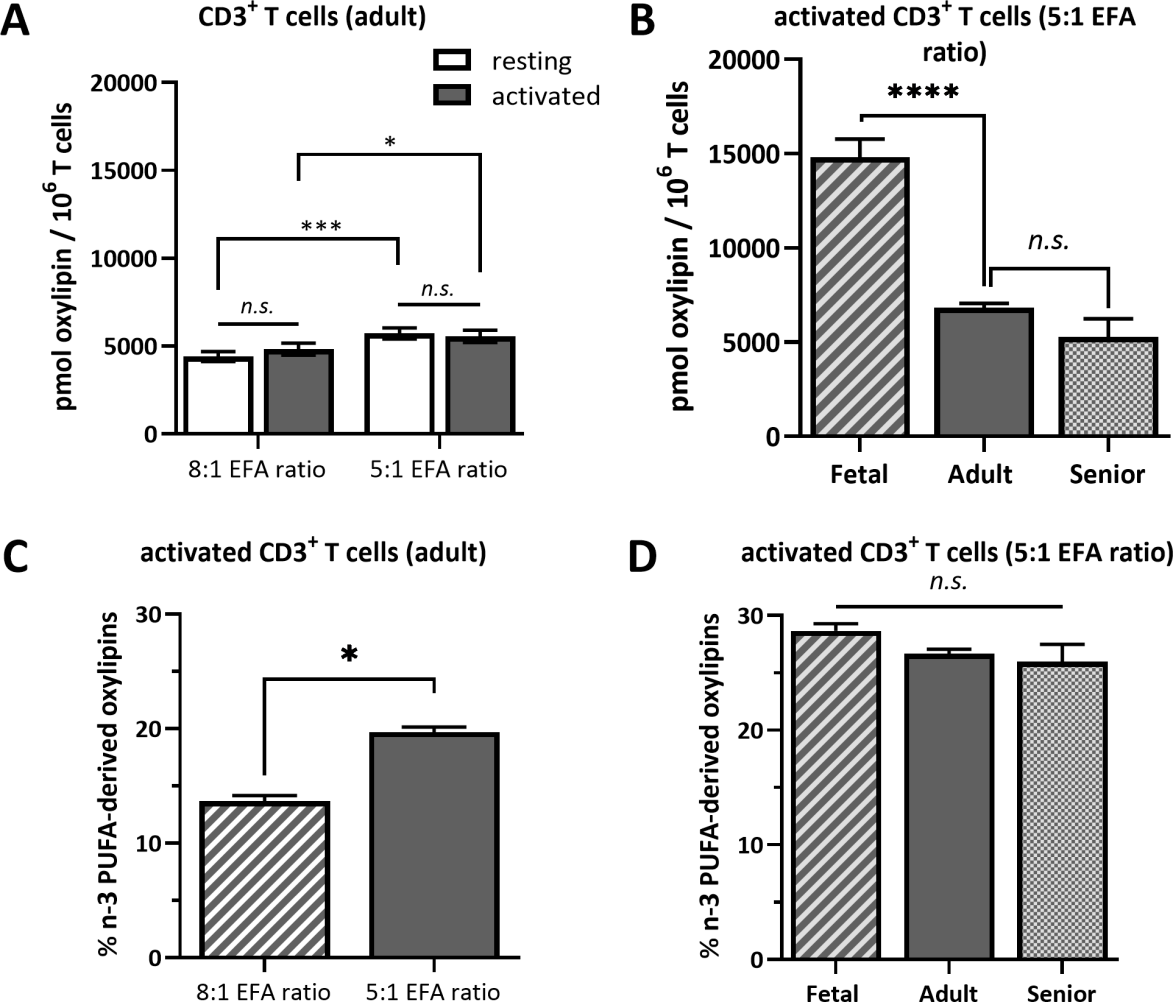
Total oxylipin concentration in the supernatant of **(A)** resting and activated adult CD3+ T cells cultured for 48 h with a 5:1 or 8:1 EFA ratio and **(B)** in the supernatant of activated T cells from fetal, adult and senior donors cultured with a 5:1 EFA ratio. The percentage of n-3 PUFA-derived oxylipins detected in the supernatant of **(C)** 48 h activated adult CD3+ T cells comparing a 5:1 versus 8:1 EFA ratio and **(D)** of activated T cells from fetal, adult and senior donors incubated with a 5:1 EFA ratio. Values are mean ± SEM. Unpaired and two-tailed T-tests (n=10 subjects (fresh blood donations) for A and C; n=8 subjects per age group (commercial) for B and D; α=0.05), *<0.05, ***<0.001 and ****<0.0001. n.s., not significant.

ALA-derived oxylipins were more abundant in supernatants from cultures using a 5:1 compared to an 8:1 EFA ratio. 9-HOTrE was increased 5-fold (P<0.001) with the 5:1 EFA ratio, 13-HOTrE 4-fold (P<0.001), 9,10-DiHODE 6-fold (P<0.001), 12,13-DiHODE 9-fold (P<0.001) and 15,16-DiHODE 5-fold (P<0.001) compared to the 8:1 EFA ratio (**Table 1**, **Figure 4**). EPA-derived oxylipin concentrations in culture supernatants from activated cultures with a 5:1 EFA ratio were also increased with 11-HEPE and 18-HEPE 2-fold (P=0.001 and P=0.002, respectively), and 11,12-DiHETE 5-fold (P<0.001) higher compared to supernatants from cultures with the 8:1 EFA ratio. The concentration of DHA-derived 17-HDHA was 1.4-fold higher (P=0.012) in supernatants from activated T cell cultures with an EFA ratio of 5:1 compared to an EFA ratio of 8:1. LA-derived 9-HODE and 13-HODE concentrations were both 1.6-fold higher (P=0.001 and P=0.004, respectively) in supernatants from activated T cell cultures containing the 5:1 compared to an 8:1 EFA ratio (**Table 1**, **Figure 4**). LA-derived 9,10-DiHOME and 12,13-DiHOME concentrations were 30-fold (P<0.001) and 32-fold (P<0.001) higher, in supernatants from cultures with the 8:1 compared to a 5:1 EFA ratio (**Table 1**, **Figure 4**). AA-derived LTB_4_ concentration was 38-fold higher (P<0.001) in supernatants from activated T cell cultures with the 8:1 compared to a 5:1 EFA ratio. The total amount of oxylipins, as the sum of 47 detected compounds, increased with the 5:1 EFA ratio 1.5-fold and 1.3-fold for resting and activated T cell cultures, respectively, compared to cultures incubated with the 8:1 EFA ratio (**Figure 5A**). There was no difference between resting and activated T cell cultures. The amount of n-3 PUFA-derived oxylipins was greater in the supernatant of T cell cultures with the 5:1 EFA ratio compared to an 8:1 EFA ratio (20% ± 0.5 vs 14% ± 0.5; P<0.001) (**Figure 5C**). 5,6-DiHETE concentration is 1.6-fold (P=0.001) higher with a 5:1 EFA ratio compared to supernatants from cultures with the 8:1 EFA ratio.

### Effect of life stage on individual oxylipins in supernatants from cultures of mitogen activated T lymphocytes incubated in medium containing a 5:1 EFA ratio

**Figure 6:**
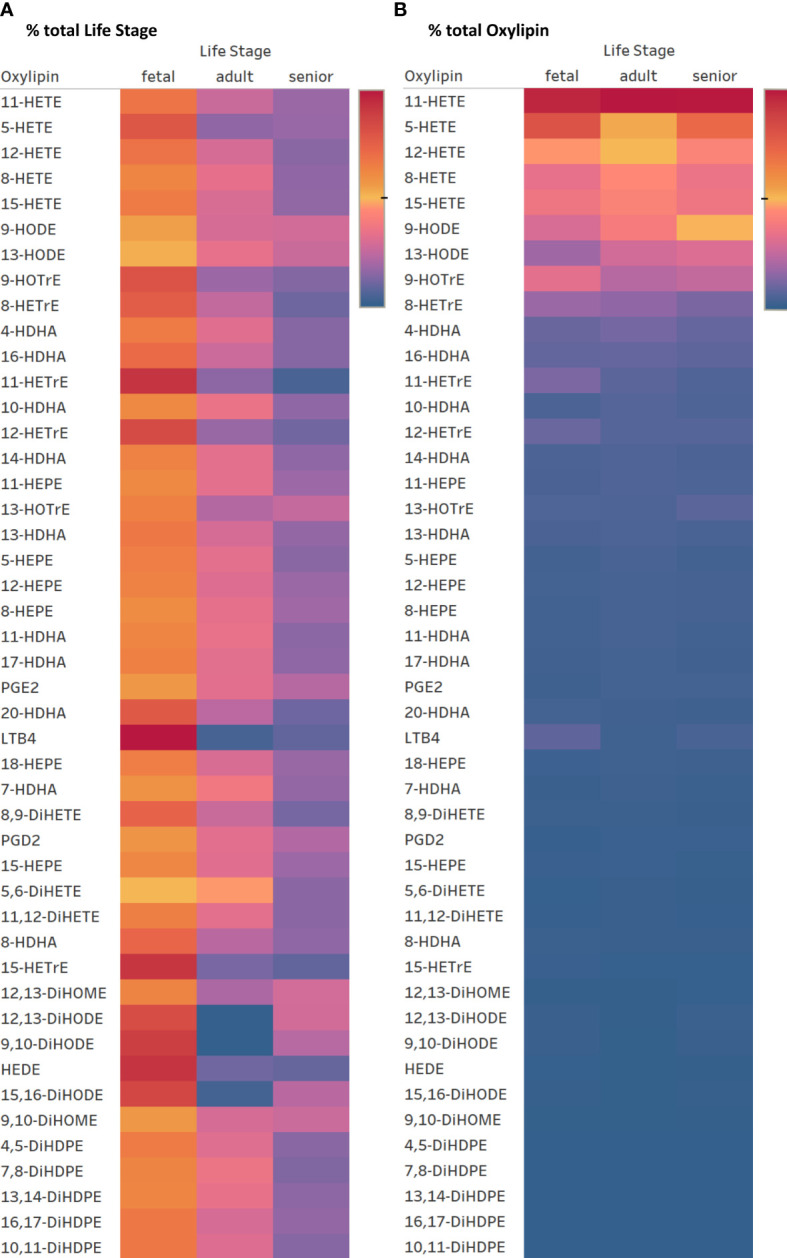
Heatmaps for oxylipin distribution in percent normalized to the total concentration of oxylipin per row = total life stage **(A)** and normalized to total oxylipin concentration per column = total oxylipin **(B)**. Color gradient indicates highest values in red over medium in yellow to lowest in blue. (2022 Tableau Desktop Software).

The same forty-seven oxylipins were detected in cultures of activated fetal T cells isolated from umbilical cord blood as well as of T cell cultures from senior adults. 11-HETE (>19.5%), 5-HETE (>10.7%) and 12-HETE (>9.2%) were the three most abundant oxylipins in fetal, adult and senior supernatants (**Supplementary Table 4**). Only two oxylipins, namely 5-HETE and HEDE, differed significantly in concentration between resting and activated cultures, whilst the concentration of ten oxylipins were affected by an interaction between activation x life stage comparing resting and activated cultures from all three life stages (fetal, adult and senior). Twenty-nine oxylipins differed significantly in concentration between life stages whilst six showed no difference in concentration (**Supplementary Table 3**). **Figure 6A** shows an overview of the difference in oxylipin concentrations in activated T cell cultures from fetal, adult and senior donors. The values for individual oxylipins were normalized to the total amount of the individual oxylipin per life stage. The total amount of oxylipins, calculated from the sum of 47 detected compounds was 2-fold higher in activated T cell cultures from fetal compared to adult donors, and there was no significant difference in total oxylipin concentration between activated cultures of T cells from seniors and adults (**Figure 5B**). The amount of n-3 PUFA-derived oxylipins was the same in the supernatant of T cell cultures from fetal, adult and senior donors (**Figure 5D**). In contrast, **Figure 6B** shows that the pattern of oxylipins was not significantly affected by the life stages, showing a similar % total oxylipin distribution comparing T cell cultures from fetal, adult and senior donors. The most abundant oxylipins were AA-derived HETEs followed by the EFA-derived HODEs and HOTrEs. These data are summarized in **Supplementary Tables 4**, **5**.

## Discussion

The present findings show that the EFA ratio of the extracellular environment has a greater effect than mitogen activation on the pattern of oxylipins secreted by adult CD3^+^ T cells. Increasing the ALA concentration in the culture medium, and therefore reducing the EFA ratio, induced broad changes in the pattern of oxylipins derived from a variety of PUFAs with a general shift towards n-3 PUFA-derived oxylipins. Furthermore, although the total oxylipin concentration in the culture supernatant was higher for T cells from umbilical cord blood, the pattern of oxylipins achieved by the 5:1 EFA ratio did not differ significantly between life stages.

The results also show that incubating human CD3^+^ T cells with different EFA ratios is reflected in a change of cellular LA to ALA ratio after 48 h (**Figure 1**). This is in agreement with previously published results from CD3^+^ T cells using stable isotope tracers that showed the uptake of EFAs added to culture medium into T cells, as well as the conversion of EFAs into 20 carbon PUFAs and oxidation into 18 carbon oxylipins (7, 15). We also show in previous studies that the concanavalin A stimulation is sufficient to activate primary T cells (7).

The main drivers for the change based on EFA ratio (VIP score, **Table 1**, **Figures 3**, **4**) were unsurprisingly the LA- and ALA-derived oxylipins as well as the AA-derived LTB_4_. The lower concentrations of n-6 EFA/longer chain n-6 PUFA-derived DiHOMEs and LTB_4_ together with higher concentrations of n-3 EFA/longer chain n-3 PUFA-derived HOTrEs, DiHODEs, HEPEs and 17-HDHA, respectively, reflects a less-inflammatory oxylipin profile in the media of adult T cells cultured with the 5:1 EFA ratio compared to the 8:1 ratio. EPA- and DHA-derived oxylipins, notably the specialized pro-resolving mediators, have been demonstrated to play a role in resolving inflammatory processes and their precursors 18-HEPE and 17-HDHA were more abundant in cultures with the lower EFA ratio (25). Although human T cells have a limited capacity to synthesize longer chain PUFAs such as AA, EPA and DHA from EFAs (7), incubation in medium containing different relative amounts of EFAs indicates that there is a shift in the pattern of oxylipins derived from these PUFAs. One possible explanation for the effect of EFAs on the pattern of oxylipins is that competition between newly assimilated EFAs for fatty acid oxidases and oxygenases displaces longer chain PUFAs from oxidation pathways in a manner related to the first double bond from the methyl end; the important n-3 (omega-3) locus.

The shift of the oxylipin profile when changing the relative exposure to the two EFA ratios is consistent with previous findings that showed a change from AA-derived HETEs to EPA-derived HEPEs in plasma from mice fed ALA-enriched butter (37) as well as increased HEPE and HDHA concentrations in plasma from healthy men on a high-ALA diet (38) (**Figures 4**, **5C**). A cell culture study using human macrophages described increased levels of ALA-derived oxylipins following ALA treatment, but also increased levels of various oxylipins from similar precursor PUFAs as shown in this study (9). Treating cells with changing levels of LA and ALA directly affects their oxidation products and high DiHOME concentrations in plasma are associated with cytotoxicity, oxidative stress and obesity-induced low grade inflammation (39, 40). The functions of HOTrE and DiHODE are still not clear, although there is some evidence that HOTrE can have beneficial functions such as reducing lipid droplet accumulation in adipocytes (41) and anti-inflammatory effects on macrophages through NLRP3 inactivation (42, 43). Furthermore, we suggested previously that LA and ALA compete for oxidation by lipoxygenases and cytochromes with a preference for ALA when provided in a higher concentration (5:1 EFA ratio). Since EFAs and other PUFAs all compete for the same set of oxidative enzymes, this could be due to cellular levels of substrate or substrate preference of the individual enzymes. However, a limitation of this study is that we cannot determine which oxylipins are formed enzymatically and which are formed by autoxidation and hence limiting the interpretation of this data regarding T cell enzymatic substrate preference.

Treating activated cultures of T cells from umbilical cord blood and from peripheral blood from senior donors with the 5:1 EFA ratio provides a model to study the metabolism of different T cell subsets. CD3^+^ T cells isolated from umbilical cord blood are mainly naïve T cells (44) whereas those extracted from peripheral blood of adults are a mixture of naïve, memory and effector T cells with more memory and effector cells in seniors than younger adults (45). However, despite the likely presence of different T cell subsets in the different life stages, we found the relative composition of oxylipins conserved across the life stages when exposing the cells to the same EFA ratio (**Figures 5D**, **6**). The results indicate that the proportion of n-3 PUFA-derived oxylipins induced by the higher ALA concentration is consistent over the life stages suggesting that a less-inflammatory oxylipin profile is present throughout the life course when the EFA ratio is lower. Although the pattern of oxylipins in supernatants of T cells treated with the same EFA ratio is similar from donors at different life stages, the total amount of oxylipins secreted into the supernatant differs. The absolute amount of oxylipins in the cell supernatant of fetal T cells is approximately double that of T cells from adults or senior adults suggesting a much higher metabolism/oxidation of presented EFAs by fetal T cells. A higher capacity in synthesis and secretion of oxylipins by fetal T cells may be linked to their higher immune tolerance and the lower production of oxylipins in later life stages might be a contributing factor to immunosenescence.

The percent composition of oxylipins reveals that the majority of them show no difference between T cell cultures from fetal, adult, and senior donors (**Figure 6**). However, in T cell cultures of fetal as well as senior donors ALA-derived DiHODEs showed a noticeable higher proportion compared to T cell cultures from adults. The roles that these DiHODEs play in immune function are yet to be determined.

The beneficial health effects that come with lowering the n-6:n-3 ratio through dietary intake is reflected in the European Food Safety Authority (EFSA) recommendations for EPA and DHA of 250 mg/day to maintain normal cardiac function (46). Others have shown beneficial effects for mental health and prevention of cognitive decline due to an increase of n-3 PUFAs in the diet (47, 48) and during the SARS-CoV-2 pandemic evidence for the inflammation resolving capacity of dietary n-3 PUFA metabolites, namely oxylipins, emerged (49–51). The findings of this study suggest that an increase in dietary ALA that changes an EFA ratio similar to the UK’s (8:1) to an EFA ratio more similar to for example Denmark’s (5:1) (33) may have beneficial effects on human immune function by promoting the release of n-3 PUFA-derived anti-inflammatory oxylipins, including those formed from EPA and DHA. Further work could evaluate the effects of EFA treatment on other immunoregulatory effects eg cytokine production.

Here, we demonstrate that the less inflammatory oxylipin profile of T cells from adult donors treated with a higher ALA concentration (5:1 EFA ratio) is largely stable over life stages implying that the change in flux of PUFA oxidation induced by varying the amount of EFAs is not affected by age. Furthermore, we interpreted some of our findings to suggest that the capacity to produce oxylipins is higher for naïve T cells than for memory and effector T cells, as reflected by the higher amount of total oxylipins in supernatants of cultured T cells from umbilical cord blood. These findings have implications for dietary recommendations for LA and ALA to promote healthy immune ageing and for understanding health benefits of plant-based diets (52).

## Data availability statement

The original contributions presented in the study are included in the article/**Supplementary Material**. Further inquiries can be directed to the corresponding author.

## Ethics statement

The studies involving human participants were reviewed and approved by East of England - Cambridge Central Research Ethics Committee; approval number 19/EE/0096. The patients/participants provided their written informed consent to participate in this study.

## Author contributions

GB, BF, PC, EM, and KL conceived and designed the study. JG, AW, and NI carried out the experiments and, together with BF and GB, analyzed the data. JG wrote the first draft of the article. All authors contributed to the article and approved the submitted version.
