# Supplementary material for: Oxylipin secretion by human CD3+ T lymphocytes in vitro is modified by the exogenous essential fatty acid ratio and life stage
Source: Front Immunol. 2023 Jun 14;14:1206733. doi: 10.3389/fimmu.2023.1206733 (PMC10300345; doi:10.3389/fimmu.2023.1206733)
Supplement: Supplementary file 1 [file DataSheet_1.docx]

Supplementary Table 1 Multiple reaction monitoring (MRM) transitions (Q1>Q3), limit of detection (LOD), limit of quantification (LOQ), SPE Recovery and inter-assay coefficient of variation (CV) of oxylipins measured with LC-MS/MS.

| -derived | Chemical Name | Oxylipin | Q1 mass [m/z] | Q3 masses [m/z] | CE [eV] | LOD / LOQ [ng/mL] | % SPE recovery | Inter-assay %CV |
| --- | --- | --- | --- | --- | --- | --- | --- | --- |
| Internal  standards | trihydroxy- | PGF1a-d9 | 364.3 | 302 | 25 | - | 82 | 16 |
|  | dihydroxy- | 14,15-DiHETrE-d11 | 348.3 | 207 | 15 | - | - | 14 |
|  | hydroxy- | 17-HDHA-d5 | 348.3 | 201/286 | 15 | - | 86 | 20 |
| 18:2n-6 | hydroxyoctadecaenoic acid | 9-HODE | 295.2 | 171/233 | 25 | 3.17 / 9.61 | 92 | 18 |
| (LA) |  | 13-HODE | 295.2 | 195/251 | 25 | - | - | - |
|  | dihydroxyoctadecanoic acid | 9,10-DiHOME | 313.3 | 201/277 | 25 | - | - | - |
|  |  | 12,13-DiHOME | 313.3 | 183/295 | 25 | - | - | - |
| 20:2n-6 | hydroxyeicosaenoic acid | 11-HEDE | 323.3 | 171/199 | 14 | - | - | - |
| (EDA) |  | 12-HEDE | 323.3 | 183 | 14 | - | - | - |
|  |  | 15-HEDE | 323.3 | 223 | 14 | - | - | - |
|  |  | X-HEDE | 323.3 | 181/221/305 | 14 | - | - | - |
|  | dihydroxyeicosaenoic acid | X,X-DiHEE | 341.3 | 279/297/303/323 | 14 | - | - | - |
|  | dihydroxyeicosadienoic | 11,14-DiHEDE | 339.3 | 227 | 18 | - | - | - |
|  | acid | 12,15-DiHEDE | 339.3 | 211 | 18 | - | - | - |
| 20:3n-6 | hydroxyeicosatrienoic acid | 8-HETrE | 321.3 | 157 | 15 | - | - | - |
| (DGLA) |  | 9-HETrE | 321.3 | 125 | 15 | - | - | - |
|  |  | 11-HETrE | 321.3 | 169 | 15 | - | - | - |
|  |  | 12-HETrE | 321.3 | 181 | 15 | - | - | - |
|  |  | 15-HETrE | 321.3 | 177 | 15 | - | - | - |
|  | dihydroxyeicosadienoic | 8,9-DiHEDE | 339.3 | 141/159/187 | 18 | - | - | - |
|  | acid | 11,12-DiHEDE | 339.3 | 181/183 | 18 | - | - | - |
|  |  | 14,15-DiHEDE | 339.3 | 221/223/237 | 18 | - | - | - |
|  |  | X,X-DiHEDE | 339.3 | 277/295/321 | 18 | - | - | - |
|  | dihydroxyeicosatrienoic | 8,X-DiHETrE | 337.3 | 129/157 | 16 | - | - | - |
|  | acid | X,11-DiHETrE | 337.3 | 195/185/213 | 16 | - | - | - |
|  |  | 12,X-DiHETrE | 337.3 | 181/209 | 16 | - | - | - |
|  |  | X,15-DiHETrE | 337.3 | 237/265 | 16 | - | - | - |
|  |  | X,X-DiHETrE | 337.3 | 275/319 | 16 | - | - | - |
|  | prostaglandin | PGF1α | 355.3 | 293 | 25 | - | - | - |
|  |  | PGE1 | 353.3 | 235/317 | 18 | - | - | - |
|  | thromboxane | TxB1 | 371.3 | 171 | 15 | - | - | - |
| 20:4n-6 | hydroxyeicosatetraenoic | 5-HETE | 319.2 | 115 | 14 | 3.45 / 10.47 | 100 | 12 |
| (AA) | acid | 8-HETE | 319.2 | 155 | 14 | 4.61 / 13.93 | 92 | 7 |
|  |  | 9-HETE | 319.2 | 123 | 14 | - | - | - |
|  |  | 11-HETE | 319.2 | 167 | 14 | 2.36 / 7.15 | 100 | 7 |
|  |  | 12-HETE | 319.2 | 179 | 14 | 3.58 / 10.85 | 100 | 10 |
|  |  | 15-HETE | 319.2 | 219 | 14 | 3.01 / 9.13 | 100 | 7 |
|  |  | 16-HETE | 319.2 | 189 | 14 | - | - | - |
|  |  | 17-HETE | 319.2 | 247 | 14 | - | - | - |
|  |  | 18-HETE | 319.2 | 261 | 14 | - | - | - |
|  |  | 19-HETE | 319.2 | 231 | 14 | - | - | - |
|  |  | 20-HETE | 319.2 | 245 | 14 | - | - | - |
|  | dihydroxyeicosatrienoic | 5,6-DiHETrE | 337.3 | 115/145 | 16 | - | - | - |
|  | acid | 8,9-DiHETrE | 337.3 | 127 | 16 | - | - | - |
|  |  | 11,12-DiHETrE | 337.3 | 167 | 16 | - | - | - |
|  |  | 14,15-DiHETrE | 337.3 | 207 | 16 | - | - | - |
|  | dihydroxyeicosatetraenoic | LTB4 (5,12-DiHETE) | 335.2 | 129/195 | 14 | 2.62 / 7.95 | 75 | 7 |
|  | acid | 5,15-DiHETE | 335.2 | 235/263 | 14 | - | - | - |
|  |  | 8,15-DiHETE | 335.2 | 169 | 14 | - | - | - |
|  |  | X,X-DiHETE | 335.2 | 273/317 | 14 | - | - | - |
|  | trihydroxyeicosatetraenoic | LxA4 | 351.2 | 115/217/235 | 17 | - | - | - |
|  | acid | LxB4 | 351.2 | 221/233/251 | 17 | - | - | - |
|  | prostaglandin | PGF2α | 353.3 | 193 | 23 | 3.36 / 10.19 | 88 | 10 |
|  |  | PGE2 | 351.2 | 271 | 18 | 4.07 / 12.32 | 92 | 5 |
|  |  | PGD2 | 351.2 | 271 | 18 | 4.04 / 12.23 | 89 | 10 |
|  |  | 6-keto-PGF1α | 369.3 | 163 | 25 | 3.56 / 10.79 | 83 | 23 |
|  | thromboxane | TxB2 | 369.3 | 269 | 15 | 5.49 / 16.64 | 93 | 10 |
| 18:3n-3 | hydroxyoctadecatrienoic | 9-HOTrE | 293.2 | 171/249 | 23 | - | - | - |
| (ALA) | acid | 13-HOTrE | 293.2 | 195/231 | 23 | - | - | - |
|  | dihydroxyoctadecadienoic | 9,10-DiHODE | 311.2 | 201 | 25 | - | - | - |
|  | acid | 12,13-DiHODE | 311.2 | 183 | 25 | - | - | - |
|  |  | 15,16-DiHODE | 311.2 | 223 | 25 | - | - | - |
|  |  | X-DiHODE | 311.2 | 275/293 | 25 | - | - | - |
| 20:5n-3 | hydroxyeicosapentaenoic | 5-HEPE | 317.2 | 115 | 14 | - | - | - |
| (EPA) | acid | 8-HEPE | 317.2 | 155 | 14 | - | - | - |
|  |  | 9-HEPE | 317.2 | 149 | 14 | - | - | - |
|  |  | 11-HEPE | 317.2 | 167 | 14 | - | - | - |
|  |  | 12-HEPE | 317.2 | 179 | 14 | - | - | - |
|  |  | 15-HEPE | 317.2 | 175 | 14 | - | - | - |
|  |  | 16-HEPE | 317.2 | 189 | 14 | - | - | - |
|  |  | 17-HEPE | 317.2 | 203 | 14 | - | - | - |
|  |  | 18-HEPE | 317.2 | 215 | 14 | - | - | - |
|  |  | 19-HEPE | 317.2 | 229 | 14 | - | - | - |
|  |  | 20-HEPE | 317.2 | 243 | 14 | - | - | - |
|  |  | X-HEPE | 317.2 | 255/273 | 14 | - | - | - |
|  | dihydroxyeicosatetraenoic | 5,6-DiHETE | 335.2 | 115 | 14 | - | - | - |
|  | acid | 8,9-DiHETE | 335.2 | 127 | 14 | - | - | - |
|  |  | 11,12-DiHETE | 335.2 | 167 | 14 | - | - | - |
|  |  | 14,15-DiHETE | 335.2 | 207 | 14 | - | - | - |
|  |  | 17,18-DiHETE | 335.2 | 247 | 14 | - | - | - |
|  | dihydroxyeicosapentaenoic | LTB5 | 333.2 | 195 | 14 | - | - | - |
|  | acid |  |  |  |  | - | - | - |
|  | trihydroxyeicosapentaenoic acid | LxA5 | 349.2 | 115/215 | 17 | - | - | - |
|  | prostaglandin | PGF3α | 351.2 | 193 | 23 | - | - | - |
|  |  | PGE3 | 349.2 | 269/313 | 18 | - | - | - |
|  | thromboxane | TxB3 | 367.2 | 169 | 15 | - | - | - |
|  | resolvin | RvE1 | 349.2 | 195 | 17 | 2.85 / 8.63 | 89 | 12 |
|  |  | RvE2 | 333.2 | 253 | 17 | - | - | - |
|  |  | RvE3 | 333.2 | 201 | 17 | - | - | - |
| 22:6n-3 | hydroxydocosahexaenoic | 4-HDHA | 343.2 | 101 | 14 | - | - | - |
| (DHA) | acid | 7-HDHA | 343.2 | 141 | 14 | - | - | - |
|  |  | 8-HDHA | 343.2 | 109 | 14 | - | - | - |
|  |  | 10-HDHA | 343.2 | 153 | 14 | - | - | - |
|  |  | 11-HDHA | 343.2 | 149 | 14 | - | - | - |
|  |  | 13-HDHA | 343.2 | 193 | 14 | - | - | - |
|  |  | 14-HDHA | 343.2 | 161 | 14 | - | - | - |
|  |  | 16-HDHA | 343.2 | 233 | 14 | - | - | - |
|  |  | 17-HDHA | 343.2 | 201 | 14 | - | - | - |
|  |  | 20-HDHA | 343.2 | 241 | 14 | - | - | - |
|  | dihydroxydocosapentaenoic | 4,5-DiHDPE | 361.3 | 73/101 | 14 | - | - | - |
|  | acid | 7,8-DiHDPE | 361.3 | 113/141 | 14 | - | - | - |
|  |  | 10,11-DiHDPE | 361.3 | 153/181 | 14 | - | - | - |
|  |  | 13,14-DiHDPE | 361.3 | 193/221 | 14 | - | - | - |
|  |  | 16,17-DiHDPE | 361.3 | 233/261 | 14 | - | - | - |
|  |  | 19,20-DiHDPE | 361.3 | 229/273 | 14 | - | - | - |
|  |  | X-DiHDPE | 361.3 | 299/343 | 14 | - | - | - |
|  | resolvin | RvD1 | 375.2 | 141 | 17 | - | - | - |
|  |  | RvD2 | 375.2 | 175 | 17 | - | - | - |
|  |  | RvD3 | 375.2 | 147 | 17 | - | - | - |
|  |  | RvD4 | 375.2 | 101 | 17 | - | - | - |
|  |  | RvD5 | 375.2 | 199 | 17 | - | - | - |
|  |  | RvD6 | 375.2 | 101 | 17 | - | - | - |
|  | maresin | MaR1 | 359.2 | 177/221/250 | 17 | - | - | - |
|  | protectin | PDX | 359.2 | 153/205 | 17 | - | - | - |
|  |  | (N)PD1 | 359.2 | 153/206 | 17 | - | - | - |

Supplementary Table 2 Fold change as log (pmol oxylipin per 10^6^ T cells) of oxylipins in supernatant of adult donor T cell cultures with an 8:1 and 5:1 EFA ratio. 15 corresponding VIP compounds reported in table 1.

| pmol oxylipin per 10^6^ T cells | log (5:1/8:1 EFA ratio) | | | | | | P values | | | | |
| --- | --- | --- | --- | --- | --- | --- | --- | --- | --- | --- | --- |
|  | Resting | | | Activated | | | Activation | EFA ratio | Activation x EFA ratio | Resting 5:1 vs 8:1 | Activated 5:1 vs 8:1 |
| 16,17-DiHDPE | 0.224 | ± | 0.025 | 0.143 | ± | 0.012 | 0.855 | 0.001 | 0.099 | 0.001 | 0.006 |
| 10,11-DiHDPE | 0.204 | ± | 0.020 | 0.152 | ± | 0.013 | 0.393 | 0.002 | 0.201 | <0.001 | 0.017 |
| 10-HDHA | 0.176 | ± | 0.012 | 0.035 | ± | 0.003 | 0.241 | 0.032 | 0.057 | 0.004 | 0.588 |
| 11-HDHA | 0.183 | ± | 0.012 | 0.091 | ± | 0.006 | 0.392 | 0.013 | 0.185 | 0.007 | 0.164 |
| 11-HEPE | 0.198 | ± | 0.031 | 0.326 | ± | 0.036 | 0.779 | <0.001 | 0.140 | <0.001 | 0.001 |
| 11-HETE | 0.104 | ± | 0.008 | 0.022 | ± | 0.002 | 0.321 | 0.161 | 0.128 | 0.027 | 0.703 |
| 11-HETrE | 0.181 | ± | 0.013 | 0.044 | ± | 0.004 | <0.001 | 0.039 | 0.015 | 0.004 | 0.507 |
| 12-HEPE | 0.197 | ± | 0.028 | 0.258 | ± | 0.022 | 0.418 | 0.003 | 0.472 | 0.018 | <0.001 |
| 12-HETE | 0.128 | ± | 0.008 | 0.046 | ± | 0.004 | 0.520 | 0.051 | 0.062 | 0.006 | 0.379 |
| 12-HETrE | 0.183 | ± | 0.015 | 0.016 | ± | 0.001 | 0.027 | 0.026 | 0.001 | 0.001 | 0.737 |
| 13,14-DiHDPE | 0.216 | ± | 0.023 | 0.089 | ± | 0.018 | 0.875 | 0.035 | 0.158 | <0.001 | 0.357 |
| 13-HDHA | 0.193 | ± | 0.012 | 0.092 | ± | 0.006 | 0.077 | 0.003 | 0.060 | 0.003 | 0.048 |
| 14-HDHA | 0.218 | ± | 0.017 | 0.093 | ± | 0.006 | 0.247 | 0.001 | 0.093 | 0.0025 | 0.070 |
| 15-HEPE | 0.204 | ± | 0.028 | 0.369 | ± | 0.041 | 0.861 | 0.014 | 0.319 | 0.139 | 0.011 |
| 15-HETE | 0.114 | ± | 0.011 | 0.124 | ± | 0.010 | 0.522 | 0.013 | 0.793 | 0.009 | 0.052 |
| 15-HETrE | 0.191 | ± | 0.041 | -0.039 | ± | 0.007 | 0.003 | 0.218 | 0.088 | 0.055 | 0.776 |
| 16-HDHA | 0.191 | ± | 0.015 | 0.168 | ± | 0.013 | 0.855 | 0.004 | 0.744 | 0.004 | 0.022 |
| 20-HDHA | 0.142 | ± | 0.011 | 0.091 | ± | 0.005 | 0.849 | 0.030 | 0.431 | 0.014 | 0.168 |
| 4,5-DiHDPE | 0.311 | ± | 0.057 | -0.072 | ± | 0.0134 | 0.258 | 0.238 | 0.016 | 0.015 | 0.647 |
| 4-HDHA | 0.285 | ± | 0.025 | -0.039 | ± | 0.003 | 0.007 | 0.036 | 0.001 | <0.001 | 0.629 |
| 5-HEPE | 0.253 | ± | 0.032 | 0.146 | ± | 0.015 | 0.757 | <0.001 | 0.208 | <0.001 | 0.022 |
| 5-HETE | 0.106 | ± | 0.006 | -0.069 | ± | 0.005 | 0.030 | 0.635 | 0.002 | 0.053 | 0.257 |
| 7,8-DiHDPE | 0.153 | ± | 0.021 | 0.177 | ± | 0.035 | 0.921 | 0.080 | 0.877 | 0.118 | 0.227 |
| 7-HDHA | 0.201 | ± | 0.016 | 0.047 | ± | 0.002 | 0.333 | 0.016 | 0.039 | 0.012 | 0.270 |
| 8,9-DiHETE | 0.140 | ± | 0.011 | 0.074 | ± | 0.005 | 0.772 | 0.025 | 0.123 | 0.007 | 0.151 |
| 8-HDHA | 0.177 | ± | 0.016 | -0.010 | ± | 0.001 | 0.017 | 0.028 | 0.010 | 0.002 | 0.843 |
| 8-HEPE | 0.053 | ± | 0.012 | 0.150 | ± | 0.015 | 0.856 | 0.227 | 0.350 | 0.633 | 0.056 |
| 8-HETE | 0.029 | ± | 0.002 | -0.025 | ± | 0.002 | 0.407 | 0.953 | 0.242 | 0.546 | 0.667 |
| 8-HETrE | 0.068 | ± | 0.005 | -0.078 | ± | 0.005 | 0.071 | 0.976 | 0.001 | 0.174 | 0.119 |
| HEDE | 0.163 | ± | 0.022 | -0.005 | ± | 0.001 | 0.269 | 0.082 | 0.253 | 0.112 | 0.941 |
| PGD2 | 0.215 | ± | 0.021 | 0.029 | ± | 0.003 | 0.109 | 0.026 | 0.031 | <0.001 | 0.644 |
| PGE2 | 0.139 | ± | 0.012 | 0.006 | ± | 0.001 | 0.077 | 0.031 | 0.098 | <0.001 | 0.884 |

Values are mean ± SEM (n = 10 paired). Comparisons were done by linear mixed model, and statistical significance was assumed at p < 0.05. Adjustment for multiple t-tests: Sidak method. EFA ratio = in the culture medium at T0.

Supplementary Table 3 Fold change as log (pmol oxylipin per 10^6^ T cells) of oxylipins in supernatants of activated T cell cultures incubated with a 5:1 EFA ratio for the life stages fetal (from umbilical cord blood), senior and adult.

| pmol oxylipin per 10^6^ T cells | log (fetal/adult) | | | | | | log (senior/adult) | | | | | | P values | | |
| --- | --- | --- | --- | --- | --- | --- | --- | --- | --- | --- | --- | --- | --- | --- | --- |
|  | Resting | | | Activated | | | Resting | | | Activated | | | Activation | Life stage | Activation x Life stage |
| 9-HODE | -0.102 | ± | 0.008 | -0.124 | ± | 0.010 | -0.023 | ± | 0.002 | 0.113 | ± | 0.009 | 0.715 | 0.036 | 0.834 |
| 13-HODE | -0.136 | ± | 0.010 | -0.186 | ± | 0.014 | -0.054 | ± | 0.004 | 0.048 | ± | 0.004 | 0.765 | 0.017 | 0.826 |
| 9,10-DiHOME | 0.187 | ± | 0.024 | -0.111 | ± | 0.014 | 0.107 | ± | 0.014 | 0.167 | ± | 0.021 | 0.067 | 0.402 | 0.096 |
| 12,13-DiHOME | 0.329 | ± | 0.032 | 0.025 | ± | 0.004 | 0.243 | ± | 0.023 | 0.286 | ± | 0.045 | 0.085 | 0.039 | 0.210 |
| LTB4 | 0.467 | ± | 0.024 | 0.509 | ± | 0.046 | 0.053 | ± | 0.003 | 0.238 | ± | 0.022 | 0.973 | 0.002 | 0.674 |
| 9-HOTrE | 0.212 | ± | 0.008 | 0.150 | ± | 0.009 | 0.136 | ± | 0.005 | 0.118 | ± | 0.007 | 0.099 | 0.131 | 0.553 |
| 13-HOTrE | 0.133 | ± | 0.008 | 0.006 | ± | 0.000 | 0.103 | ± | 0.006 | 0.210 | ± | 0.009 | 0.212 | 0.215 | 0.145 |
| 9,10-DiHODE | 0.599 | ± | 0.098 | 0.577 | ± | 0.045 | 0.690 | ± | 0.113 | 0.629 | ± | 0.049 | 0.862 | 0.001 | 0.786 |
| 12,13-DiHODE | 0.454 | ± | 0.093 | 0.533 | ± | 0.056 | 0.687 | ± | 0.141 | 0.671 | ± | 0.070 | 0.434 | 0.002 | 0.515 |
| 15,16-DiHODE | 0.453 | ± | 0.043 | 0.459 | ± | 0.029 | 0.526 | ± | 0.050 | 0.528 | ± | 0.034 | 0.275 | 0.001 | 0.842 |
| 11-HEPE | -0.054 | ± | 0.002 | -0.130 | ± | 0.009 | -0.062 | ± | 0.003 | -0.001 | ± | 0.000 | 0.544 | 0.006 | 0.259 |
| 18-HEPE | 0.025 | ± | 0.001 | -0.083 | ± | 0.006 | -0.020 | ± | 0.001 | 0.012 | ± | 0.001 | 0.737 | 0.759 | 0.302 |
| 5,6-DiHETE | -0.467 | ± | 0.031 | -0.300 | ± | 0.010 | -0.236 | ± | 0.016 | -0.150 | ± | 0.005 | 0.246 | 0.000 | 0.106 |
| 11,12-DiHETE | -0.135 | ± | 0.015 | -0.081 | ± | 0.011 | -0.121 | ± | 0.014 | -0.036 | ± | 0.005 | 0.402 | 0.194 | 0.616 |
| 17-HDHA | -0.042 | ± | 0.002 | -0.103 | ± | 0.004 | -0.102 | ± | 0.004 | -0.028 | ± | 0.001 | 0.568 | 0.010 | 0.251 |
| 16,17-DiHDPE | -0.019 | ± | 0.002 | -0.036 | ± | 0.003 | -0.106 | ± | 0.010 | -0.018 | ± | 0.002 | 0.084 | 0.733 | 0.748 |
| 10,11-DiHDPE | 0.414 | ± | 0.016 | 0.261 | ± | 0.016 | -0.366 | ± | 0.014 | -0.219 | ± | 0.013 | 0.173 | <0.001 | 0.078 |
| 10-HDHA | 0.323 | ± | 0.008 | 0.199 | ± | 0.003 | -0.248 | ± | 0.006 | -0.227 | ± | 0.003 | 0.075 | <0.001 | 0.094 |
| 11-HDHA | 0.342 | ± | 0.012 | 0.210 | ± | 0.003 | -0.257 | ± | 0.009 | -0.231 | ± | 0.004 | 0.037 | <0.001 | 0.048 |
| 11-HETE | 0.470 | ± | 0.020 | 0.322 | ± | 0.006 | -0.154 | ± | 0.006 | -0.112 | ± | 0.002 | 0.312 | 0.002 | 0.083 |
| 11-HETrE | 0.608 | ± | 0.030 | 0.582 | ± | 0.038 | -0.221 | ± | 0.011 | -0.223 | ± | 0.015 | 0.146 | <0.001 | 0.297 |
| 12-HEPE | 0.395 | ± | 0.024 | 0.246 | ± | 0.011 | -0.186 | ± | 0.011 | -0.166 | ± | 0.007 | 0.108 | 0.001 | 0.067 |
| 12-HETE | 0.423 | ± | 0.017 | 0.293 | ± | 0.004 | -0.207 | ± | 0.008 | -0.189 | ± | 0.002 | 0.137 | <0.001 | 0.123 |
| 12-HETrE | 0.593 | ± | 0.053 | 0.519 | ± | 0.049 | -0.119 | ± | 0.011 | -0.103 | ± | 0.010 | 0.073 | <0.001 | 0.170 |
| 13,14-DiHDPE | 0.434 | ± | 0.028 | 0.214 | ± | 0.006 | -0.226 | ± | 0.014 | -0.225 | ± | 0.006 | 0.015 | <0.001 | 0.012 |
| 13-HDHA | 0.429 | ± | 0.017 | 0.286 | ± | 0.008 | -0.181 | ± | 0.007 | -0.161 | ± | 0.005 | 0.115 | <0.001 | 0.041 |
| 13-HODE | 0.260 | ± | 0.027 | 0.151 | ± | 0.011 | -0.296 | ± | 0.031 | -0.373 | ± | 0.026 | 0.221 | <0.001 | 0.346 |
| 14-HDHA | 0.361 | ± | 0.008 | 0.229 | ± | 0.003 | -0.242 | ± | 0.005 | -0.211 | ± | 0.003 | 0.098 | <0.001 | 0.093 |
| 15-HEPE | 0.397 | ± | 0.027 | 0.229 | ± | 0.013 | -0.169 | ± | 0.011 | -0.166 | ± | 0.009 | 0.112 | 0.001 | 0.091 |
| 15-HETE | 0.442 | ± | 0.016 | 0.273 | ± | 0.004 | -0.179 | ± | 0.007 | -0.175 | ± | 0.003 | 0.082 | 0.001 | 0.033 |
| 15-HETrE | 0.668 | ± | 0.018 | 0.638 | ± | 0.017 | -0.088 | ± | 0.002 | -0.068 | ± | 0.002 | 0.236 | <0.001 | 0.543 |
| 16-HDHA | 0.390 | ± | 0.007 | 0.334 | ± | 0.018 | -0.140 | ± | 0.003 | -0.168 | ± | 0.009 | 0.123 | <0.001 | 0.579 |
| 20-HDHA | 0.488 | ± | 0.015 | 0.404 | ± | 0.011 | -0.148 | ± | 0.004 | -0.201 | ± | 0.005 | 0.109 | <0.001 | 0.359 |
| 4,5-DiHDPE | 0.388 | ± | 0.018 | 0.254 | ± | 0.010 | -0.281 | ± | 0.013 | -0.215 | ± | 0.008 | 0.038 | <0.001 | 0.038 |
| 4-HDHA | 0.383 | ± | 0.019 | 0.251 | ± | 0.013 | -0.239 | ± | 0.012 | -0.224 | ± | 0.012 | 0.116 | <0.001 | 0.091 |
| 5-HEPE | 0.391 | ± | 0.012 | 0.235 | ± | 0.008 | -0.242 | ± | 0.007 | -0.227 | ± | 0.008 | 0.039 | 0.001 | 0.040 |
| 5-HETE | 0.683 | ± | 0.095 | 0.516 | ± | 0.058 | 0.037 | ± | 0.005 | 0.018 | ± | 0.002 | 0.049 | <0.001 | 0.098 |
| 7,8-DiHDPE | 0.335 | ± | 0.011 | 0.198 | ± | 0.009 | -0.332 | ± | 0.011 | -0.277 | ± | 0.013 | 0.119 | <0.001 | 0.032 |
| 7-HDHA | 0.271 | ± | 0.008 | 0.157 | ± | 0.005 | -0.266 | ± | 0.008 | -0.241 | ± | 0.007 | 0.142 | <0.001 | 0.121 |
| 8,9-DiHETE | 0.369 | ± | 0.007 | 0.353 | ± | 0.022 | -0.233 | ± | 0.005 | -0.211 | ± | 0.013 | 0.152 | <0.001 | 0.427 |
| 8-HDHA | 0.509 | ± | 0.018 | 0.382 | ± | 0.005 | -0.164 | ± | 0.006 | -0.108 | ± | 0.001 | 0.097 | <0.001 | 0.041 |
| 8-HEPE | 0.364 | ± | 0.020 | 0.210 | ± | 0.009 | -0.171 | ± | 0.009 | -0.166 | ± | 0.007 | 0.105 | 0.001 | 0.085 |
| 8-HETE | 0.369 | ± | 0.013 | 0.219 | ± | 0.005 | -0.216 | ± | 0.008 | -0.211 | ± | 0.005 | 0.059 | <0.001 | 0.076 |
| 8-HETrE | 0.431 | ± | 0.005 | 0.380 | ± | 0.018 | -0.229 | ± | 0.003 | -0.222 | ± | 0.011 | 0.187 | <0.001 | 0.471 |
| HEDE | 0.746 | ± | 0.043 | 0.664 | ± | 0.052 | 0.068 | ± | 0.004 | -0.032 | ± | 0.003 | 0.045 | <0.001 | 0.147 |
| PGD2 | 0.369 | ± | 0.032 | 0.198 | ± | 0.012 | -0.104 | ± | 0.009 | -0.117 | ± | 0.007 | 0.411 | <0.001 | 0.027 |
| PGE2 | 0.379 | ± | 0.031 | 0.193 | ± | 0.010 | -0.116 | ± | 0.009 | -0.108 | ± | 0.005 | 0.110 | <0.001 | 0.013 |

Values are mean ± SEM (n =8 unpaired). Comparisons were done by linear mixed model with all life stages (fetal, adult, and senior) as fixed factor, and statistical significance was assumed at p < 0.05. Adjustment for multiple t-tests: Sidak method.

Supplementary Table 4 Percent of oxylipins in supernatants normalised to total amount of oxylipins in all life stages of activated T cell cultures incubated with a 5:1 EFA ratio for the life stages fetal, senior and adult.

|  | % total Life Stage of oxylipin in supernatant of activated T cells | | | | | | | | | P values | |
| --- | --- | --- | --- | --- | --- | --- | --- | --- | --- | --- | --- |
| Life Stage: | Fetal | | | Adult | | | Senior | | | Fetal vs Adult | Adult vs Senior |
| 10,11-DiHDPE | 0.0013 | ± | 0.0001 | 0.0007 | ± | 0.0000 | 0.0004 | ± | 0.0001 | >0.9999 | >0.9999 |
| 10-HDHA | 0.5200 | ± | 0.0289 | 0.3292 | ± | 0.0048 | 0.1953 | ± | 0.0253 | 0.583 | 0.583 |
| 11,12-DiHETE | 0.1182 | ± | 0.0150 | 0.0687 | ± | 0.0107 | 0.0414 | ± | 0.0076 | 0.9754 | 0.9754 |
| 11-HDHA | 0.3082 | ± | 0.0183 | 0.1899 | ± | 0.0029 | 0.1115 | ± | 0.0153 | 0.8113 | 0.8113 |
| 11-HEPE | 0.4694 | ± | 0.0374 | 0.2842 | ± | 0.0159 | 0.1902 | ± | 0.0265 | 0.6012 | 0.648 |
| 11-HETE | 10.9165 | ± | 0.9779 | 5.2015 | ± | 0.0980 | 4.0153 | ± | 0.6697 | <0.0001 | <0.0001 |
| 11-HETrE | 1.4170 | ± | 0.1161 | 0.3710 | ± | 0.0243 | 0.2222 | ± | 0.0342 | <0.0001 | 0.47 |
| 12,13-DiHODE | 0.1463 | ± | 0.0170 | 0.0197 | ± | 0.0022 | 0.0619 | ± | 0.0116 | 0.9018 | 0.9018 |
| 12,13-DiHOME | 0.0510 | ± | 0.0082 | 0.0222 | ± | 0.0036 | 0.0274 | ± | 0.0067 | 0.9986 | 0.9986 |
| 12-HEPE | 0.3453 | ± | 0.0284 | 0.1962 | ± | 0.0087 | 0.1338 | ± | 0.0187 | 0.7181 | 0.7619 |
| 12-HETE | 5.1620 | ± | 0.3609 | 2.6289 | ± | 0.0347 | 1.7007 | ± | 0.1819 | <0.0001 | <0.0001 |
| 12-HETrE | 1.0608 | ± | 0.0864 | 0.3214 | ± | 0.0303 | 0.2537 | ± | 0.0567 | 0.0007 | 0.7423 |
| 13,14-DiHDPE | 0.0062 | ± | 0.0003 | 0.0038 | ± | 0.0001 | 0.0022 | ± | 0.0004 | >0.9999 | >0.9999 |
| 13-HDHA | 0.4707 | ± | 0.0289 | 0.2439 | ± | 0.0069 | 0.1682 | ± | 0.0218 | 0.4683 | 0.7132 |
| 13-HODE | 2.0550 | ± | 0.1469 | 1.4519 | ± | 0.1011 | 0.6153 | ± | 0.0781 | 0.0035 | 0.0001 |
| 13-HOTrE | 0.5837 | ± | 0.0357 | 0.2618 | ± | 0.0097 | 0.2871 | ± | 0.0679 | 0.3144 | 0.9022 |
| 14-HDHA | 0.4870 | ± | 0.0296 | 0.2874 | ± | 0.0037 | 0.1769 | ± | 0.0225 | 0.5544 | 0.5915 |
| 15,16-DiHODE | 0.1043 | ± | 0.0100 | 0.0165 | ± | 0.0010 | 0.0376 | ± | 0.0050 | 0.964 | 0.964 |
| 15-HEPE | 0.1253 | ± | 0.0099 | 0.0740 | ± | 0.0042 | 0.0505 | ± | 0.0079 | 0.9772 | 0.9772 |
| 15-HETE | 4.0859 | ± | 0.2912 | 2.1808 | ± | 0.0342 | 1.4576 | ± | 0.1716 | <0.0001 | 0.0005 |
| 15-HETrE | 0.1257 | ± | 0.0080 | 0.0290 | ± | 0.0008 | 0.0248 | ± | 0.0045 | 0.9469 | 0.9837 |
| 16,17-DiHDPE | 0.0015 | ± | 0.0001 | 0.0008 | ± | 0.0001 | 0.0005 | ± | 0.0001 | >0.9999 | >0.9999 |
| 16-HDHA | 0.9469 | ± | 0.0586 | 0.4386 | ± | 0.0241 | 0.2977 | ± | 0.0494 | 0.0272 | 0.4938 |
| 17-HDHA | 0.2885 | ± | 0.0165 | 0.1662 | ± | 0.0035 | 0.1042 | ± | 0.0141 | 0.7998 | 0.7998 |
| 18-HEPE | 0.2176 | ± | 0.0226 | 0.1180 | ± | 0.0061 | 0.0822 | ± | 0.0110 | 0.883 | 0.883 |
| 20-HDHA | 0.3507 | ± | 0.0226 | 0.1382 | ± | 0.0037 | 0.0871 | ± | 0.0138 | 0.5131 | 0.804 |
| 4,5-DiHDPE | 0.0070 | ± | 0.0004 | 0.0039 | ± | 0.0002 | 0.0024 | ± | 0.0005 | >0.9999 | >0.9999 |
| 4-HDHA | 1.0399 | ± | 0.0816 | 0.5838 | ± | 0.0305 | 0.3485 | ± | 0.0654 | 0.0532 | 0.2533 |
| 5,6-DiHETE | 0.0777 | ± | 0.0042 | 0.0719 | ± | 0.0030 | 0.0333 | ± | 0.0064 | 0.995 | 0.995 |
| 5-HEPE | 0.3401 | ± | 0.0261 | 0.1978 | ± | 0.0070 | 0.1172 | ± | 0.0154 | 0.7395 | 0.7395 |
| 5-HETE | 9.1128 | ± | 0.5861 | 2.7769 | ± | 0.3095 | 2.8942 | ± | 0.3471 | <0.0001 | 0.5689 |
| 7,8-DiHDPE | 0.0061 | ± | 0.0003 | 0.0039 | ± | 0.0002 | 0.0021 | ± | 0.0004 | >0.9999 | >0.9999 |
| 7-HDHA | 0.1524 | ± | 0.0101 | 0.1062 | ± | 0.0033 | 0.0609 | ± | 0.0101 | 0.969 | 0.9685 |
| 8,9-DiHETE | 0.1965 | ± | 0.0154 | 0.0873 | ± | 0.0054 | 0.0536 | ± | 0.0105 | 0.8656 | 0.87 |
| 8-HDHA | 0.1645 | ± | 0.0119 | 0.0683 | ± | 0.0009 | 0.0532 | ± | 0.0087 | 0.9305 | 0.9415 |
| 8-HEPE | 0.3119 | ± | 0.0225 | 0.1921 | ± | 0.0082 | 0.1309 | ± | 0.0175 | 0.807 | 0.807 |
| 8-HETE | 3.7798 | ± | 0.2314 | 2.2854 | ± | 0.0528 | 1.4045 | ± | 0.1554 | <0.0001 | <0.0001 |
| 8-HETrE | 1.9390 | ± | 0.1590 | 0.8084 | ± | 0.0386 | 0.4847 | ± | 0.0724 | <0.0001 | 0.1162 |
| 9,10-DiHODE | 0.1529 | ± | 0.0211 | 0.0186 | ± | 0.0017 | 0.0524 | ± | 0.0087 | 0.8854 | 0.8854 |
| 9,10-DiHOME | 0.0270 | ± | 0.0042 | 0.0161 | ± | 0.0021 | 0.0150 | ± | 0.0035 | 0.9999 | 0.9999 |
| 9-HODE | 3.3200 | ± | 0.2935 | 2.0146 | ± | 0.1542 | 0.8783 | ± | 0.1260 | <0.0001 | <0.0001 |
| 9-HOTrE | 3.6447 | ± | 0.5424 | 1.1624 | ± | 0.0503 | 1.0061 | ± | 0.0775 | <0.0001 | 0.4479 |
| HEDE | 0.0796 | ± | 0.0054 | 0.0173 | ± | 0.0014 | 0.0161 | ± | 0.0040 | 0.9858 | 0.9954 |
| LTB4 | 0.8614 | ± | 0.1355 | 0.1226 | ± | 0.0101 | 0.1562 | ± | 0.0724 | 0.001 | 0.8704 |
| PGD2 | 0.1180 | ± | 0.0079 | 0.0748 | ± | 0.0046 | 0.0571 | ± | 0.0106 | 0.9874 | 0.9874 |
| PGE2 | 0.2496 | ± | 0.0183 | 0.1601 | ± | 0.0081 | 0.1247 | ± | 0.0234 | 0.9053 | 0.9053 |

**Supplementary Table 5** Percent of oxylipins in supernatants normalised to total amount of oxylipins of activated T cell cultures incubated with a 5:1 EFA ratio for the life stages fetal, senior and adult.

|  | % oxylipin in supernatant of activated T cells | | | | | | | | | P values | |
| --- | --- | --- | --- | --- | --- | --- | --- | --- | --- | --- | --- |
| Life Stage: | Fetal | | | Adult | | | Senior | | | Fetal vs Adult | Adult vs Senior |
| 10,11-DiHDPE | 0.0024 | ± | 0.0002 | 0.0028 | ± | 0.0002 | 0.0024 | ± | 0.0004 | >0.9999 | >0.9999 |
| 10-HDHA | 0.9295 | ± | 0.0517 | 1.2750 | ± | 0.0187 | 1.0709 | ± | 0.1386 | 0.9372 | 0.9416 |
| 11,12-DiHETE | 0.2113 | ± | 0.0268 | 0.2663 | ± | 0.0413 | 0.2272 | ± | 0.0414 | 0.999 | 0.999 |
| 11-HDHA | 0.5509 | ± | 0.0328 | 0.7357 | ± | 0.0112 | 0.6113 | ± | 0.0839 | 0.989 | 0.989 |
| 11-HEPE | 0.8390 | ± | 0.0669 | 1.1008 | ± | 0.0617 | 1.0427 | ± | 0.1452 | 0.971 | 0.971 |
| 11-HETE | 19.5126 | ± | 1.7479 | 20.1478 | ± | 0.3795 | 22.0168 | ± | 3.6720 | 0.338 | 0.01 |
| 11-HETrE | 2.5329 | ± | 0.2076 | 1.4370 | ± | 0.0942 | 1.2183 | ± | 0.1876 | 0.188 | 0.742 |
| 12,13-DiHODE | 0.2615 | ± | 0.0304 | 0.0762 | ± | 0.0086 | 0.3392 | ± | 0.0638 | 0.99 | 0.99 |
| 12,13-DiHOME | 0.0912 | ± | 0.0146 | 0.0858 | ± | 0.0141 | 0.1502 | ± | 0.0366 | 0.99 | 0.99 |
| 12-HEPE | 0.6173 | ± | 0.0507 | 0.7599 | ± | 0.0335 | 0.7337 | ± | 0.1024 | 0.99 | 0.99 |
| 12-HETE | 9.2267 | ± | 0.6450 | 10.1827 | ± | 0.1345 | 9.3253 | ± | 0.9975 | 0.386 | 0.386 |
| 12-HETrE | 1.8960 | ± | 0.1545 | 1.2448 | ± | 0.1175 | 1.3913 | ± | 0.3111 | 0.695 | 0.825 |
| 13,14-DiHDPE | 0.0111 | ± | 0.0005 | 0.0146 | ± | 0.0004 | 0.0123 | ± | 0.0022 | >0.9999 | >0.9999 |
| 13-HDHA | 0.8414 | ± | 0.0517 | 0.9445 | ± | 0.0266 | 0.9221 | ± | 0.1196 | 0.99 | 0.99 |
| 13-HODE | 3.6731 | ± | 0.2626 | 5.6240 | ± | 0.3917 | 3.3739 | ± | 0.4280 | 0.007 | 0.002 |
| 13-HOTrE | 1.0433 | ± | 0.0639 | 1.0142 | ± | 0.0374 | 1.5743 | ± | 0.3724 | 0.965 | 0.783 |
| 14-HDHA | 0.8705 | ± | 0.0530 | 1.1131 | ± | 0.0142 | 0.9698 | ± | 0.1235 | 0.98 | 0.98 |
| 15,16-DiHODE | 0.1864 | ± | 0.0178 | 0.0640 | ± | 0.0040 | 0.2064 | ± | 0.0273 | 0.99 | 0.99 |
| 15-HEPE | 0.2239 | ± | 0.0177 | 0.2866 | ± | 0.0164 | 0.2770 | ± | 0.0434 | 0.99 | 0.99 |
| 15-HETE | 7.3032 | ± | 0.5205 | 8.4471 | ± | 0.1323 | 7.9925 | ± | 0.9409 | 0.234 | 0.509 |
| 15-HETrE | 0.2247 | ± | 0.0144 | 0.1122 | ± | 0.0030 | 0.1359 | ± | 0.0249 | 0.99 | 0.99 |
| 16,17-DiHDPE | 0.0027 | ± | 0.0002 | 0.0030 | ± | 0.0002 | 0.0030 | ± | 0.0007 | >0.9999 | >0.9999 |
| 16-HDHA | 1.6926 | ± | 0.1047 | 1.6990 | ± | 0.0934 | 1.6321 | ± | 0.2710 | 0.99 | 0.99 |
| 17-HDHA | 0.5157 | ± | 0.0294 | 0.6439 | ± | 0.0136 | 0.5716 | ± | 0.0775 | 0.99 | 0.99 |
| 18-HEPE | 0.3889 | ± | 0.0404 | 0.4569 | ± | 0.0237 | 0.4506 | ± | 0.0605 | 0.99 | 0.99 |
| 20-HDHA | 0.6269 | ± | 0.0404 | 0.5353 | ± | 0.0143 | 0.4774 | ± | 0.0758 | 0.99 | 0.99 |
| 4,5-DiHDPE | 0.0126 | ± | 0.0008 | 0.0152 | ± | 0.0006 | 0.0131 | ± | 0.0026 | >0.9999 | >0.9999 |
| 4-HDHA | 1.8588 | ± | 0.1459 | 2.2612 | ± | 0.1183 | 1.9112 | ± | 0.3588 | 0.905 | 0.905 |
| 5,6-DiHETE | 0.1389 | ± | 0.0076 | 0.2784 | ± | 0.0117 | 0.1823 | ± | 0.0349 | 0.99 | 0.99 |
| 5-HEPE | 0.6079 | ± | 0.0466 | 0.7663 | ± | 0.0270 | 0.6427 | ± | 0.0846 | 0.99 | 0.99 |
| 5-HETE | 16.2886 | ± | 1.0476 | 10.7562 | ± | 1.1988 | 15.8695 | ± | 1.9035 | <0.0001 | <0.0001 |
| 7,8-DiHDPE | 0.0110 | ± | 0.0005 | 0.0151 | ± | 0.0007 | 0.0113 | ± | 0.0021 | >0.9999 | >0.9999 |
| 7-HDHA | 0.2724 | ± | 0.0180 | 0.4115 | ± | 0.0127 | 0.3341 | ± | 0.0554 | 0.99 | 0.99 |
| 8,9-DiHETE | 0.3512 | ± | 0.0276 | 0.3380 | ± | 0.0207 | 0.2941 | ± | 0.0575 | 0.99 | 0.99 |
| 8-HDHA | 0.2940 | ± | 0.0213 | 0.2647 | ± | 0.0036 | 0.2919 | ± | 0.0475 | >0.9999 | >0.9999 |
| 8-HEPE | 0.5574 | ± | 0.0403 | 0.7442 | ± | 0.0318 | 0.7180 | ± | 0.0962 | 0.99 | 0.99 |
| 8-HETE | 6.7562 | ± | 0.4136 | 8.8522 | ± | 0.2047 | 7.7014 | ± | 0.8523 | 0.005 | 0.159 |
| 8-HETrE | 3.4659 | ± | 0.2843 | 3.1315 | ± | 0.1495 | 2.6577 | ± | 0.3973 | 0.725 | 0.725 |
| 9,10-DiHODE | 0.2733 | ± | 0.0376 | 0.0720 | ± | 0.0065 | 0.2874 | ± | 0.0476 | 0.99 | 0.99 |
| 9,10-DiHOME | 0.0482 | ± | 0.0076 | 0.0623 | ± | 0.0082 | 0.0825 | ± | 0.0192 | >0.9999 | >0.9999 |
| 9-HODE | 5.9343 | ± | 0.5246 | 7.8034 | ± | 0.5973 | 4.8159 | ± | 0.6909 | 0.01 | <0.0001 |
| 9-HOTrE | 6.5147 | ± | 0.9695 | 4.5026 | ± | 0.1949 | 5.5170 | ± | 0.4248 | 0.007 | 00.237 |
| HEDE | 0.1423 | ± | 0.0096 | 0.0669 | ± | 0.0053 | 0.0881 | ± | 0.0218 | 0.99 | 0.99 |
| LTB4 | 1.5397 | ± | 0.2423 | 0.4750 | ± | 0.0393 | 0.8564 | ± | 0.3970 | 0.292 | 0.566 |
| PGD2 | 0.2109 | ± | 0.0141 | 0.2896 | ± | 0.0177 | 0.3133 | ± | 0.0582 | 0.99 | 0.99 |
| PGE2 | 0.4461 | ± | 0.0327 | 0.6202 | ± | 0.0314 | 0.6840 | ± | 0.1284 | 0.98 | 0.98 |
